# Advances in biological functions and mechanisms of histone variants in plants

**DOI:** 10.3389/fgene.2023.1229782

**Published:** 2023-07-31

**Authors:** Xi Wu, Xu Zhang, Borong Huang, Junyou Han, Huihui Fang

**Affiliations:** ^1^ Jilin Province Engineering Laboratory of Plant Genetic Improvement, College of Plant Science, Jilin University, Changchun, China; ^2^ Developmental Biology, Laboratory of Plant Molecular and Zhejiang A & F University, Hangzhou, China

**Keywords:** histone variants, epigenetic regulation, biological function, growth, development, stress response

## Abstract

Nucleosome is the basic subunit of chromatin, consisting of approximately 147bp DNA wrapped around a histone octamer, containing two copies of H2A, H2B, H3 and H4. A linker histone H1 can bind nucleosomes through its conserved GH1 domain, which may promote chromatin folding into higher-order structures. Therefore, the complexity of histones act importantly for specifying chromatin and gene activities. Histone variants, encoded by separate genes and characterized by only a few amino acids differences, can affect nucleosome packaging and stability, and then modify the chromatin properties. Serving as carriers of pivotal genetic and epigenetic information, histone variants have profound significance in regulating plant growth and development, response to both biotic and abiotic stresses. At present, the biological functions of histone variants in plant have become a research hotspot. Here, we summarize recent researches on the biological functions, molecular chaperons and regulatory mechanisms of histone variants in plant, and propose some novel research directions for further study of plant histone variants research field. Our study will provide some enlightens for studying and understanding the epigenetic regulation and chromatin specialization mediated by histone variant in plant.

## 1 Introduction

Over the long evolutionary process, nucleosome has always been a defining unique feature of eukaryotes. Nucleosome, the most basic structural unit of chromatin, is consisting of ∼147 bp DNA wrapped around a histone octamer, two copies of H2A, H2B, H3, and H4. Besides these core histones, the linker histones H1, which binds to the nucleosome core at a ratio of one per nucleosome to form the chromosomes and play a role in compacting chromatin into higher order structures. This integral regulatory unit is indispensable in almost every DNA template involving processes, and the complexity, involving modifications and variants, of these histones code unquestionably has important significances on genome architecture and gene regulation (Foroozani et al., 2022).

The chromatin of plants exhibits a wide range of sequence variants of the core and linker histones. In plants, except for H4, the remaining histones H2A, H2B, H3 and linker H1 all exist in multiple variants forms that are encoded by different genes and distinguished by different protein sequences. Histone variants, acting as non-allelic protein isoforms of canonical histones, can antagonize deposition of canonical histones at specific chromatin loci to maintain stability of nucleosome and confer chromatin structure more diversity and flexibility (Martire and Banaszynski, 2020; Kurumizaka et al., 2021; Nunez-Vazquez et al., 2022; Probst, 2022). Additionally, variant protein can be modified by specific post-translational modifications (PTMs), defines distinct chromatin states that impact specific chromatin functions (Foroozani et al., 2022; Jiang and Berger, 2023). Increasing studies have indicated that histone variants influence in transcription and epigenetic states, chromosome segregation, DNA damage repair (Foroozani et al., 2022; Jiang and Berger, 2023). Overall, histone variants exhibit important transcriptional programming functions during developmental process and stress responses in plants.

In this review, we concisely summarize the researches about plant histone variants and mainly focus on their biological functions and regulatory mechanisms in transcriptional regulation during plant growth and development, environmental stress responses. Moreover, we also discuss the limitation in current researches, and propose new directions for researches in the field of plant histone variants.

## 2 Histone variants in plants

In plants, except for histone H4, all other core histones (H2A, H2B, and H3) and linker H1 have various variant forms, and most researches focus on variants of H2A and H3. H3.3 and H2A.Z are evolutionarily conserved throughout eukaryotes (Yelagandula et al., 2014; Giaimo et al., 2019). In addition, there are some lineage-specific and tissue-specific variants, such as H2A.W variants that only function in flowering plants (Bourguet et al., 2021; Lei et al., 2021), while the H3.10 and H2B.8 only existing in sperm cells of Arabidopsis (Borg et al., 2020; Jiang et al., 2020; Borg et al., 2021; Buttress et al., 2022). Species and tissues specificity endow histone variants with distinct functions. Cause of distinct amino acid sequences from the canonical histones, variant proteins exhibit distinct chromatin deposition pattern. For example, H2A.Z and H2A.X are more biased to be concentrated in the euchromatic region (Lei and Berger, 2020; Borg et al., 2021), while H2A.W is mainly located in the concentrated heterochromatin region, and different histone variants can endow nucleosomes and chromatin with unique properties. The combination of different histone variants can form hundreds or thousands different types of nucleosomes, and this flexible pattern confer great potential for regulating various physiological processes in plants by the epigenetic code. Histone variants can influence nucleosome stability, histone modification, DNA repair and methylation, transcriptional activity, epigenetic states, and other nuclear processes that profoundly involve in plant growth and development, environmental stress responses.

There is a wide variety of histone variants in plants and their physiological functions are sophisticated, therefore, in the next paragraph, histone variants will be classified according to their family, and the similarities and differences between different variants in the same family will be summarized, which will provide reference for further research on the biological functions of histone variants in plant. In Arabidopsis, the majority of histones variants are organized in Table 1 discussed below.

**TABLE 1:** Classification of histone variant family, including coding gene loci, chaperone, related generally physiological functions and roles in development and stress responses in Arabidopsis.

Histone variants		Gene loci	Chaperone	General function	Roles in stress response, growth and development
H2A	H2A.X	AT1G54690	FACT?	Transcriptional activation, DNA damage repair	
		AT1G08880			
	H2A.W	AT5G59870	DDM1	Maintain the silence of heterochromatin	
		AT5G27670		Chromatin compaction, DNA damage repair	
		AT5G02560			
	H2A.Z	AT2G38810	SWR1	Transcriptional activation and repression	Flowering time Deal et al. (2007); Sijacic et al. (2019); vegetative phase change Gómez-Zambrano et al. (2018); Xu et al. (2018); Inflorescence architecture Cai et al. (2019); Salt, drought, extreme temperature, phosphate deficiency and immunity responses; Germline development Zhao et al. (2018); Circadian clock Tong et al. (2020)
		AT1G52740	MBD9		
		AT3G54560	INO80		
H2B	H2B.1	AT1G07790	NAP1		
			NRP1		
			FACT		
	H2B.2	AT5G22880			
	H2B.3	AT2G28720			
	H2B.4	AT5G59910			
	H2B.5	AT2G37470			
	H2B.6	AT3G53650			
	H2B.7	AT3G09480			
	H2B.8	AT1G08170			Regulate seed development but not expressed in sperm cells
	H2B.9	AT3G45980			
	H2B.10	AT5G02570			
	H2B.11	AT3G46030			
H3	H3.3	AT4G40030	HIRA	Transcriptional activation	Flowering time Zhao et al. (2021); Male Gametogenesis Ingouff et al. (2010); Cell proliferation and organogenesis Otero et al. (2016)
		AT4G40040	ATRX		
		AT5G10980			
	H3.6	AT1G13370	HIRA?		
	H3.7	AT1G75610			
	H3.10	AT1G19890	HIRA?		Regulate epigenetic reprogramming of Arabidopsis spermatocytes
	H3.11	AT5G65350			
	CenH3	AT1G01370	KNL2	Involved in the formation of spindle during cell division	induce haploid production
			NASP		
	H3.14	AT1G75600	HIRA?		
	H3.15	AT5G12910	HIRA		Promote callus formation and tissue regeneration

### 2.1 H2A variants

The H2A histone variants, playing vital roles in numerous biological processes, are widely studied. The three most widely studied histone variants of H2A are H2A.Z, H2A.X, and H2A.W, among them, H2A.W is specific in flowering plant, whereas H2A.X and H2A.Z are evolutionarily conserved among different species. Different H2A variants are distinguished mainly by three main features: L1 loop, docking domain and C-terminal tail.

The H2A.Z variant has a shorter C-terminal tail, which is a typical feature that differs from canonical H2A. The C-terminal tail of H2A.Z contains a KD/E conservative motif, and L1 loop contains a conservative S/TAHG motif, and the H2A.Z docking domain differs from other H2A histone variants. In Arabidopsis, there are a total of three genes encoding H2A proteins. The HTA8/9/11, which are redundant but exhibit different expression patterns, are responsible for encoding H2A.Z (Yi et al., 2006; Sijacic et al., 2019). In *Arabidopsis*, most active genes contain a prominent H2A.Z peak at the +1 nucleosome beyond the transcription start site (TSS) (Foroozani et al., 2022), while H2A.Z is mainly enriched in the gene body region of genes with low expression. H2A.Z is deposited into nucleosome by the ATP dependent SWR1 complex (SWI2/SNF2 related 1 complex) (Verbsky and Richards, 2001; Kobor et al., 2004; Mizuguchi et al., 2004), and the core subunits of SWR1 include PIE1 (PHOTOPERIOD-INDE PENDENT EARLY FLOWERING 1), ARP6 (ACTIN RELATED PROTEIN 6), SWC4 (SWR COMPLEX SUBUNIT 4) and SWC6 (SWR COMPLEX SUBUNIT 6) (Kobor et al., 2004; Mizuguchi et al., 2004; March-Díaz et al., 2007; Carter et al., 2018; Potok et al., 2019). Recent studies have shown that MBD9 (methy-CpG-binding domain 9) (Sijacic et al., 2019), YAF9 (yeast all1-fused gene from chromosome 9) (Crevillén et al., 2019) also participated in the assembly of H2A.Z into nucleosomes. On the contrary, NRP1 (NAP1 related protein 1) and NRP2 perform opposite function. Arabidopsis NRP1 and NRP2 proteins interacting with H2A.Z are also involved in the removal of H2A.Z from nucleosome in a SWR1 complex dependent manner, thus preventing the excessive accumulation of H2A.Z in nucleosome (Wang et al., 2020). INO80 control sliding and displacing of the canonical histone H2A and the variant H2A (Eustermann et al., 2018).

**FIGURE 1:**
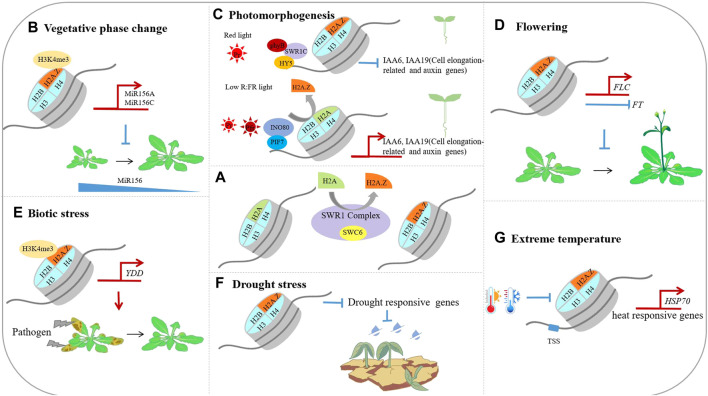
The biological functions of the histone variants in *Arabidopsis thaliana*. **(A)**. H2A.Z is deposited into nucleosome by the ATP dependent SWR1 complex; **(B)**. H2A.Z facilitates the deposition of H3K4me3 on MIR156A/MIR156C loci, and maintains a high level of MIR156A/MIR156C transcriptions; **(C)**. Under red light conditions, the SWR1 complex deposit H2A.Z at auxin and cell elongation-related genes such as IAA6 and IAA19 to repress their expression, resulting in photomorphogenesis. Under low R∶FR light condition, the INO80 complex mediate H2A.Z eviction from PIF leading to shade avoidance response; **(D)**. H2A.Z facilitates the transcription of FLC and inhibit the expression of FT, and prevent plants from entering reproductive stage early; **(E)**. H2A.Z facilitates the deposition of H3K4me3 on YDD, thereby contributing the plants tolerance to Sclerotinia sclerotiorum; **(F)**. H2A.Z facilitates the expression of drought responsive genes; **(G)**. H2A.Z facilitates the expression of heat responsive genes such as HSP70.

H2A.Z occupied a high proportion of total H2A content in cells, and involved many chromatins mediated processes, including changes of chromatin state as well as transcriptional activation or repression. In terrestrial plants, most H2A.Z-substituted nucleosomes are involved in the repression of transcription. H2A.Z inhibits transcriptional activity by promoting the recruitment of H3K27me3—a repressive histone modification, and preventing the deposition of H3K4me3, an active histone modification, thereby repressing gene transcription (Sequeira-Mendes et al., 2014; Yelagandula et al., 2014). Similar to conventional histones, histone variant H2A. Z could also be post-translationally modified, and the mono-ubiquitin modification of 129th lysine residue in H2A.Z by the PRC1 is also related to transcriptional repression (Gómez-Zambrano et al., 2019). Overall, H2A is involved in regulating all aspects of plant growth and development, and mutants loss of H2A function cause pleiotropic phenotypes including early vegetative phase change, early flowering, reduced fertility and others. In Arabidopsis, the biological functions of H2A.Z are organized in Figure 1 below.

(4) The transcription of genes regulated by histone variants involves a variety of epigenetic modifications, such as H3K4me3, H3K27me3, and histone variants co-localize with epigenetic modifications in the same genomic regions, thus they work together to influence the chromatin landscape, but it is unclear how the corresponding regulatory factors cooperate to achieve this transcription regulation.

Unlike H2A.Z, H2A.X variant has great similarity with H2A in terms of amino acid sequence, and the subtle feature that distinguishes H2A.X from canonical H2A is the presence of an SQEF motif at the C-terminal of H2A.X. H2A.X is enriched primarily in euchromatin and bodies of expressed genes (Yelagandula et al., 2014; Lorković et al., 2017). The histone chaperone of H2A.X is FACT (Facilitate Chromatin Transcription) complex in mammalian, while the assembly mechanism of H2A.X in plants has not been revealed. However, based on the fact that FACT chaperone is conserved in eukaryotes, it is reasonable to speculate that FACT may perform a similar function in plants (Foroozani et al., 2022). The most classic and characteristic function of H2A.X is to participate in the repair of DNA damage (Redon et al., 2011), which mainly depend on the phosphorylation of SQEF motif. When DNA is damaged, the serine residue of the SQEF motif in H2A.X is phosphorylated and then repair factors is recruited to repair the DNA damage (Bewersdorf et al., 2006).

The H2A.W variant is exclusive to the flowering plant (Kawashima et al., 2015). Particularly, the expression of *H2A.W* is restricted to S-phase, and its deposition is replication dependent (Gómez-Zambrano et al., 2019). H2A.W synergizes with the heterochromatin marker H3K9me2 and mainly distributed in the heterochromatin region, and H2A.W can promote heterochromatin condensation thus maintaining heterochromatin silence state (Yelagandula et al., 2014). The deposition of H2A.W alters the chromatin properties, therefore, preventing transposable elements (TEs) mobility mediated by DDM1 (DECREASE IN DNA METHYLATION 1) and leading to the silencing of TEs (Osakabe et al., 2021). At the same time, H2A.W can antagonize excessive H1 incorporation on heterochromatin, maintaining appropriate both CG and non-CG methylation level on the transposon (Bourguet et al., 2021). H2A.W has the same function as H2A.X in DNA damage repair. When DNA is damaged, the serine residue of the SQ motif in C-terminal of H2A.W. is phosphorylated by ATM (Ataxia-telangiectasia mutated kinase), thus playing an essential role in promoting DNA damage repair in the heterochromatin region (Lorković et al., 2017).

### 2.2 H2B variants

Compared with the well characterized histone variants of other core histones, the understanding of variants of H2B is relatively limited. The H2B variants of plants have undergone substantial evolutionary divergence, and the sequence between different H2B variants are diverse (Jiang et al., 2020). The H2B.S is highly expressed in sperm cells and mature embryo cells, both of which have highly concentrated chromatin. H2B.S variant specifically accumulates in dry seeds of several flowering plants. It indicates that the assembly of H2B.S may be beneficial to the concentration of chromatin. However, the study of H2B variants in plants is still in inception phase, and their specific functions need to be deeply studied in the future.

### 2.3 H3 variants

In plants, H3 has three main variants, H3.1, H3.3, and centromeric H3 variants (CenH3/CENP-A). Among them, H3.3 and CenH3 variants are most widely studied. Unlike other histone variant family, H3 family has high amino acid homology in convergent evolution. Canonical H3.1 and variants H3.3 are distinguished by four different amino acids: the 31st (Ala vs. Thr), 41st (Phe vs. Tyr), 87th (Ser vs. His) and 90th (Ala vs. Leu) (Talbert et al., 2012). Despite such tiny divergence, H3.1 and H3.3 have significant differences in modification and deposition manner. The distinction of these four different amino acids facilitates H3.1 to specifically recruit PRC2 to ensure the silence state of some developmental related genes. Recently, the combination of multiple technology methods such as immunofluorescence and genome-wide ChIP-seq data revealed that H3.1 and H3.3 have significant differences in deposition manner. H3.1 is predominantly associated with transposable elements, peri-centromeric heterochromatin and heterochromatin domains on chromosome arms, while H3.3 is enriched at the transcription end sites (TES) of genes (Stroud et al., 2012; Wollmann et al., 2012). A recent research shows that the variation of 41st amino acid (Phe vs. Tyr) partially contributes to the different distribution of H3.1 and H3.3 (Lu et al., 2018). Although H3.3 is predominantly enriched at the TES and associated with activated histone modification marks such as H3K4me3, H3K9me3, and H3K36me3, H3.3 does not affect global transcription but specifically affects the genes in response to biotic and abiotic stresses (Wollmann et al., 2017). H3.1 is mainly expressed in the S phase of the cell cycle, while H3.3 can be deposited through all stages of cell cycle in a DNA replication independent manner (Ahmad and Henikoff, 2002; Tagami et al., 2004). As the specific molecular chaperone of H3.1, CAF1 (chromatin assembly factor 1) can interact with DNA replication fork protein PCNA (PROLIFERATING CELL NUCLEAR ANTIGEN) to help H3.1 incorporate onto the newly synthesized nucleosome in S phase; whereas the H3.3 is transferred by ASF1 (Anti-Silencing Factor 1) to the typical chaperone HIRA (Histone regulator homolog A) complex, which can bind to 87th histidine and 90th leucine amino acids at C-terminal tail of H3.3 and get deposited through all stages of cell cycle (Daniel Ricketts et al., 2015). Since other less studied variants including H3.6, H3.14, and H3.10 all contain the 87th histidine and 90th leucine amino acids, whether HIRA also plays the role as molecular chaperone in their incorporations need to be demonstrated in future. Shorten breeding life.

The amino acid sequence and protein structure of CenH3 is very different from H3.1 and H3.3, especially at the N-terminal. CenH3 is located in the centromere region and plays key roles in centromere formation and kinetochore assembly, and the incorporation of CenH3 into nucleosomes occurs in the mitosis G2 stage (Lermontova et al., 2006; McKinley and Cheeseman, 2016). Unlike other H3 variants that have been thoroughly studied, there are few studies on the mechanism of CenH3 assembly into nucleosomes, and most studies of CenH3 are focused on its functions in shorting the breeding period. In recent years, scientists have exploited a series of techniques for haploid induction (HI) through manipulation of CenH3. HI can create true-breeding lines in a short period of time, which can greatly accelerate the pace of plant breeding (Lv et al., 2020; Aboobucker et al., 2023). At present, HI technology relying on CenH3/CENP-A transformation in wheat and maize has been reported (Lv et al., 2020; Wang N. et al., 2021).

In addition to the main variants of H3 described above, there are also some atypical H3 variants in Arabidopsis, for instance H3.15 and H3.10 while H3.15 plays an essential role in callus differentiation during plant regeneration (Yan et al., 2020), H3.10 is specially expressed in sperm and participates in the epigenetic reprogramming of spermatocytes (Borg et al., 2020).

### 2.4 Linker H1 variants

The linker H1 histone serves as a bridge connection for DNA and nucleosomes and facilitates chromatin compaction. H1 can bind to nucleosome through its binding sites in conserved GH1 domain and draw close to adjacent nucleosome to compress chromatin (Bednar et al., 2017). There are three variants of H1 in Arabidopsis: H1.1, H1.2, and H1.3. Among them, H1.1 and H1.2 are the main H1 variants donors. All of these H1 variants contain three conservative structures: a short N-terminus, GH1 central globular domain, and lysine rich C-terminus (Zhou et al., 2013). H1.3 is a histone variant induced by stresses such as low light, drought, or ABA (Rutowicz et al., 2015), and H1.3 with shorter N and C terminals and a lack of (S/T) PXK motifs that bind to DNA. All of these H1 variants are mainly enriched in the heterochromatin region and gene body, H1.1 and H1.2 are negatively correlated with gene expression and H3K4me3 deposition, while the negative correlation of H1.3 with gene expression and H3K4me3 deposition is weaker than H1.1 and H1.2 (Rutowicz et al., 2015). H1.3 is induced under stress and competes for the binding sites of H1.1 and H1.2, thus changing the accessibility of chromatin.

## 3 Biological functions of histone variants in plants

Plant growth and development is orchestrated by specific gene expression in a spatio-temporal manner. Additionally, plants also adjust their growth and metabolism in response to environmental stimuli by altering gene expression. Histone variants contribute importantly to specify the chromatin complexity and gene activities, and increasing findings on the variants reveal that they have essential functions during plant development in response to the external environment. Therefore, we summarize the important functions and the downstream mechanisms of histone variants redefining the plant chromatin landscape that plants perceive and transmit both developmental and environmental signals.

### 3.1 Roles of histone variants in plant growth and development

Flowering is an indispensable stage for higher plants, and histone variants play important roles in regulating plant flower.

H2A.Z affects flower through regulating the transcript levels of the floral repressor *FLC*. Reduction of H2A.Z enrichment near the TSS region on FLC chromatin repressing its expression and leading to premature flowering (Deal et al., 2007). In this process, FRI interacts with SWR1 complex to form a complex (FRI-C), which mediates H2A.Z enrichment at FLC chromatin loci and regulate FLC expression (Choi et al., 2011). Early flowering phenotypes were also observed in arp6, pie1, sef, yf9, mbd9 mutants, which were also associated with H2A.Z loading and deposition. The SWR1 complex (SWR1C) can catalyze the substitution of H2A to H2A.Z, and mutations in members of SWR1C, such as SWC6, SUF3 and PIE1, also lead to a series of development defective phenotypes, including leaf serration, apical dominance inhibition, flower deformity and accompanied with early flowering (Choi et al., 2007; Fan et al., 2022). Consistently, the early flowering phenomena of these mutants are all caused by the decrease of FLC expression caused by H2A.Z loading defects. Unexpectedly, nap1nap2 double mutants display an increase of H2A.Z enrichment at the TSS of FLC but a decrease of FLC expression, this finding indicated that H2A.Z enrichment at the TSS region of FLC is not prerequisite for FLC activation, and H2A.Z plays a dual role of activation and repression in gene transcription. The modification of H2A.Z also plays important roles in regulating the flower process, such as the acetylation of H2A.Z (Crevillén et al., 2019). Besides H2A.Z, H3.3 also participate in regulating floral transition in a FLC dependent pathway. H3.3 deposition and H3.3 mediated histone modifications are key controllers for flowering regulation through FLC transcription. FRI and HIRA chaperones collaborate to deposit H3.3 at the FLC locus and increase the activating histone modifications H3K4me3 and H3K36me3 at the FLC chromatin locus. Besides, the researchers discovered that H3.3 can facilitate the formation of 5′ to 3’ gene loop in FLC thereby promoting its transcriptional activation through chromosome conformation capture (3C) technology (Zhao et al., 2021). In addition to regulating flowering, H3.3 is also involved in regulating seed germination. H3.3 exhibits a seed specific 5′gene end distribution, and H3.3 is essential for proper gene transcriptional regulation during germination (Zhao et al., 2022).

In addition to regulating plant flowering in a *FLC*-dependent manner, H2A.Z could also regulate plant growth and development through some important microRNAs. H2A.Z facilitates the deposition of H3K4me3 on MIR156A/MIR156C loci, and maintains a high level of MIR156A/MIR156C transcriptions in the early stage of shoot development (Xu et al., 2018). In arp6 and pie1 mutants, reduced H2A.Z deposition and increased relative nucleosome possession of the MIR396A promoter significantly repressed miR396 expression, which accelerated plant leaf growth and vegetative phase change (Hou et al., 2019). These researches indicate that H2A.Z plays significant role in the process of plant growth and development regulated by miRNA.

In addition to regulate flower and vegetative transition, histone variants also involve in spermatocytes formation and callus differentiation. Unlike animals, specific histone variants package sperm chromatin of plants (Borg et al., 2020). H2B.8 performs a vital act in chromatin condensation of spermatocytes in flowering plants. H2B.8 (also known as H2B.S) specifically expressed in spermatocytes of flowering plants and is important for fertility. H2B.8 can specifically bind to the euchromatin transposon and the inter-gene region, condense the transcriptionally inactive region through phase separation, which facilitates nuclear compaction of the spermatocyte chromatin without affecting the gene expression (Buttress et al., 2022). In addition, variant of H3 family--H3.10 participate in the epigenetic reprogramming of sperm chromatin as well (Borg et al., 2020). H3.15 expression is rapidly induced to promote callus differentiation during plant regeneration. The replacement of lysine with histidine in the 27th amino acid of H3.15 leads to the failure of H3K27me3 deposition on the 27th amino acid mediated by the poly-comb repressive complex 2 (PRC2) on H3.15, and then the assembly of H3.15 will cause the removal of H3K27me3 from the chromatin, which will lead to transcriptional de-repression of genes related to callus formation and tissue regeneration (Yan et al., 2020).

Accurate modulation of hypocotyl cell extension is essential for plant growth and survival. Under the light, the plant presents the phenotype of the suppressed hypocotyl elongation and unfolded cotyledons, while under the dark condition, the hypocotyl elongates rapidly and cotyledons close. This process by which plants perceive the light and adjust their morphology is called photo-morphogenesis, a necessary developmental process from plant seedlings, which is jointly regulated by multiple pathways. Research has indicated that the deposition of H2A.Z is essential for the formation of photo-morphogenesis. NF-YCs (NUCLEAR FACTOR-Y) directly interact with ARP6 (ACTIN-RELATED PROTEIN6) to induce the deposition of H2A.Z in a light dependent manner. Enrichment of H2A.Z occupancy inhibits the expression of some auxin related genes such as IAA6 and IAA19, which then suppresses the growth of hypocotyls and promotes the photo-morphogenesis (Zhang et al., 2021).

PIFs direct interact with SWC6 and repress H2A.Z deposition at auxin-responsive genes such as *IAA6* and *IAA19*, thus upregulate the expression of *IAA6* and *IAA19* in a red light dependent manner (Chen et al., 2023). Except to *IAA6* and *IAA19*, H2A.Z deposited on gene body of auxin response genes *HY5* to regulated hypocotyl elongation in the INO80 dependent manner (Yang et al., 2020). Compared with the deposition or eviction mechanism of H2A.Z, the transcriptional regulation of H2A.Z is still lacking in available research (Yin et al., 2023). Recently, Fang et al. shed a form of feedback regulation of auxin signaling through the transcription and deposition of H2A.Z, and ARF7/19-HB22/25-mediated H2A.Z transcription to modulate the activation of *SAURs* (*SMALL AUXIN UP RNAs*) and plant growth in *Arabidopsis* (Sun et al., 2022).

Similar to the photo-morphogenesis, plants will adjust their morphology and architecture under high temperature, such as elongations of hypocotyl and petiole as well as early flowering, which are collectively termed as thermal morphogenesis. Thermal morphogenesis can facilitate plant to reduce their own temperature and better adapt to high-temperature environment (Qin et al., 2022).

There is considerable overlap and crosstalk between plant thermal morphogenesis and photo-morphogenesis, and H2A.Z plays indispensable role in both these two physiological processes. Contrary to photo-morphogenesis, H2A.Z plays an inhibitory role in process of plant thermal morphogenesis. When the ambient temperature rises, INO80 chromatin remodeling complex interacts with PIF4 (PHYTOCHROME-INTERACTING FACTOR 4), and mediating the expulsion of H2A.Z from nucleosomes of *PIF4* (Xue et al., 2021). INO80-C, a molecular chaperone, promotes H3K4me3 enrichment on PIF4 and enhances its transcription (Xue et al., 2021). INO80-C links H2A.Z eviction and transcription to co-modulate the expression of high temperature response associated genes, promoting the thermal morphogenesis of Arabidopsis. INO80 is an ATP-dependent chromatin remodeling factors that can establish and maintain the dynamic structure of chromatin employing the energy provided by ATP hydrolysis, and INO80 is necessary for H2A.Z loading to nucleosome of target genes. H2A.Z-INO80 module participates not only in photo morphogenesis and thermal morphogenesis, but also in the regulation of gibberellin pathway in rice. H2A.Z-INO80 module influences the GA biosynthesis pathway, and knockdown of INO80 causes decrease of H2A.Z enrichment at CPS1 and GA3ox2 loci, leading to a dwarf phenotype that can be partially restored by the spray of exogenous GA3 (Li et al., 2018).

### 3.2 Roles of histone variants in plant responses to biotic stress

In normal environments, plants maintain their growth and development accompanied by suppression of the functions of immune system. Microbial pathogens are the most significant biotic stresses to which plants are exposed, and therefore plants have evolved comprehensive immune response strategies to sense and deal with infection by viruses, bacteria, fungi, and other pathogens. With the refinement of the network between epigenetics and plant immunity, histone variants have been revealed to functions importantly in plants response to biotic stresses (Kim, 2021).

After perceiving pathogens, plants transduce the signals to alter phytohormone homeostasis and activate the transcriptions of resistance genes, ultimately initiating immune mechanisms. *Puccinia striiformis* f. sp. *tritici* (*Pst*) is a fungus that causes stripe rust in the host and reduces crop yield. Under high temperature, the TaCRK10 (Cysteine-rich receptor-like kinases 10) interacts with TaH2A.1, a variant of H2A.W, to mediate its phosphorylation and binding to related resistance genes, thereby enhancing resistance to stripe rust in wheat via the SA signaling pathway (Wang J. et al., 2021). *Sclerotinia sclerotiorum* causes white mold in plants, especially crucifers, whereas the critical element of SWR1C and the mutation of H2A.Z will affect the resistance of Arabidopsis to this fungus (Jakada et al., 2019; Cai et al., 2021). Further studies reveal that SWR1C promotes the expression of *YDD* (*YODA DOWNSTREAM*), a process that involves two epigenetic modifications, H2A.Z deposition and H3K4me3 modification, on *YDD*, thereby contributing to Arabidopsis tolerance to *S. sclerotiorum* (Cai et al., 2021). Similarly, *arp6*, *pie1* and *hta9hta11* mutants alter the disease resistance of Arabidopsis to the phytopathogenic bacteria *Pseudomonas syringae* (March-Díaz et al., 2008; Berriri et al., 2016). These results suggest that the SWR1C and histone variants regulate systemic acquired resistance in Arabidopsis. In addition, monoubiquitination of histone variants also affects plant disease resistance (Li et al., 2015). Under normal conditions, BRHIS1 (BIT-responsive Histone-interacting SNF2 ATPase 1) containing complex suppresses the expression of disease defense associated genes by interacting with histone variants H2A.Xa/H2A.Xb/H2A.3 and H2B.7, which are deposited in the promoter region of these genes. Upon pathogen infection in rice, decreased *BRHIS1* expression is accompanied by increased deposition of monoubiquitinated histone variants to initiate the immune response (Li et al., 2015).

### 3.3 Roles of histone variants in plants responses to abiotic stresses

Plant growth and crop yield are co-regulated by both internal and external conditions, whereas the increased frequency and duration of extreme environments poses challenges to plants survival, so that plants have evolved multiple complex strategies to respond to abiotic stresses such as salt stress, phosphate deficiency, drought, high temperature, *etc.* Epigenetic regulation comes into action through histone variants in plant adaptation to adversities (Wollmann et al., 2017; Halder et al., 2022). Here, we review current researches on functions of histone variants in plant responses to abiotic stress and discuss the intricate mechanisms, with the hope of providing additional valuable references for crop breeding.

Excessive accumulation and deficiency of soil salinity alters plant photosynthesis, water uptake, and metabolism, which ultimately reduces crop production (Van Zelm et al., 2020; Liu, 2021). Plants have evolved multiple molecular mechanisms such as osmotic adjustment and ionic homeostasis to mitigate the damage caused by salt stress, where epigenetic modification of chromatin presents novel perspectives (Singroha et al., 2022). After salt treatment, a salt-tolerant grapevine rootstock exhibits three-fold decrease of H2A.X expression (Çakır Aydemir et al., 2020). Under salt stress, the reduction of H2A.Z deposition at the promoter and gene body region of *AtMYB44* triggers the transcription of *AtMYB44* and enhances Arabidopsis resistance, combined with unaffected levels of histone modifications, includingH3K4me3, H3 and H4 acetylation (Nguyen et al., 2018). Loss of function of ARP6, which affects H2A.Z deposition, causes inhibition of root development in Arabidopsis subjected to salt stress (Do et al., 2023). In rice, phosphate deficiency induces a decline in H2A.Z accumulation on the gene bodies of stress-responsive genes and facilitates the expression of these genes, allowing the plant to adapt to environmental changes (Zahraeifard et al., 2018).

Water deficit delays plant growth by causing a decline in biomass accumulation, while the drought response mechanisms are complex and unrefined. During response to mild water deficit, the higher stomatal density of *h1.3* plants resulting in the enhanced photosynthetic rate and then in accelerated growth (Rutowicz et al., 2015). The increased transcription level of H1.3 contributes importantly to plant responses to drought and low light stresses (Rutowicz et al., 2015). Furthermore, H1.3 plays an irreplaceable role in maintaining redox homeostasis and stomatal development (Rutowicz et al., 2015; Probst et al., 2020). By studying the gene expression profile of Arabidopsis, it was revealed that the transcriptions of drought responsive genes are negatively correlated with the enrichments of H2A.Z in their gene body regions (Sura et al., 2017).

Extreme temperature affects the balance between development and reproduction of plants, which is also an important perspective for studying the functions of histone variants. Under high temperature, *arp6* mutant exhibits hypocotyl and petiole elongation along with increased expression of heat responsive genes and *HSP70* (*Heat shock protein 70*), and ChIP analysis reveals that the enrichment of H2A.Z on the HSP70 promoter is reduced, relieving its repressive effects on HSP70 expression (Kumar and Wigge, 2010). Further studies reveal that the INO80 complex mediates the eviction of H2A.Z and consequently activates the expressions of thermo-responsive genes, thereby contributing thermal morphogenesis of Arabidopsis (Xue et al., 2021). Heat stress regulates plant flowering time by altering the deposition of H2A.Z on FT gene, whereas this regulatory effect is specific in different plant species (Del Olmo et al., 2019; Abelenda et al., 2023). In conclusion, current researches on functions of histone variants in plants response to stresses have been focused on the H2A variants, and further investigations are needed to reveal the functions of other histone variants.

## 4 Conculsion

The dynamic properties and structural integrity of the nucleosome are crucial to maintain chromosome activity, and variants of the core canonical histone, profoundly affecting chromatin properties, play a prominent role in plant biological functions. At present, various researches are focusing on H2A.Z, especially on H2A.Z mediated transcriptional regulation, which has become a hot topic in the field of plant biology. The current research gives us a preliminary understanding of the biological functions and mechanisms of histones in plants, while there are still much explorations that need to be conducted for in-depth studies. Future researches can be explored in the following areas:

(1) As important epigenetic regulatory factors, histone variants play a key role in plant growth and development and stress responses. At present, most of the histone variant researches on plants is based on animal researches, and majority of researches in plant all focus on H2A.Z. There are many cell/tissues specific histone variants with low expression in plants, such as and H2B.S and CENH3/CENP-A, it is difficult to use conventional methods to reveal their functions. The cognitions of their biological functions are limited and still in primary exploration stage. With the continuous development of new technology such as single cell sequencing technology, CUT&Tag (Cleavage Under Target sand Tag mentation) can be applied to the study of histone variants. These technologies have the advantages of low sample input, high signal-to-noise ratio, and future research can use these technologies to clarify the molecular chaperone, depositing and evicting mechanism in distinct cell types;
(2) The assembly and deposition of histone variants undergo a dynamic process in a specific growth stage or receiving environmental stimuli signals such as salt stress, drought, extreme temperature, or nutrient deficit. And for plant cell, it is crucial to reprogram their chromosome landscape to respond to stimuli signals. What roles does epigenetic modifications play in these processes and whether there are tissue or time specific epigenetic modifications changes? The mechanism of how the molecular chaperone receive the internal or external environmental signals, thus assemble and unload the corresponding variants remain need to be intensively explored;
(3) In addition, related researches mainly focus on *Arabidopsis*, however, there are few studies relate on horticultural crops. With the deepening of research on the exploration of plant histone variants, the histone variants are expected to provide more important reference for crop breeding;
